# Combination Diuretic Therapy With Thiazides: A Systematic Review on the Beneficial Approach to Overcome Refractory Fluid Overload in Heart Failure

**DOI:** 10.7759/cureus.44624

**Published:** 2023-09-03

**Authors:** Amaresh Gogikar, Ankita Nanda, Lakshmi Sai Niharika Janga, Hembashima G Sambe, Mohamed Yasir, Ruzhual K Man, Lubna Mohammed

**Affiliations:** 1 Research, California Institute of Behavioral Neurosciences & Psychology, Fairfield, USA

**Keywords:** hfref, metolazone, thiazide, furosemide, loop diuretics, heart failure

## Abstract

Heart failure (HF) is a notable public health issue, and intravenous loop diuretics are frequently employed to address acute decompensated heart failure (ADHF) and alleviate symptoms of congestion. However, prolonged use of loop diuretics can lead to drug resistance, and some patients experience refractory volume overload that does not respond to treatment. Sequential nephron blockade, which involves combining loop and thiazide diuretics, has been proposed as a strategy to overcome diuretic resistance and improve fluid overload management. This systematic review aims to critically evaluate the effectiveness and safety of this combination diuretic therapy.

Following the directives detailed in the 2020 Preferred Reporting Items for Systematic Reviews and Meta-Analyses (PRISMA) guidelines, a comprehensive search was conducted. Eligibility criteria were established to select relevant studies, including the requirement for studies to be conducted on human subjects and published as free full-text papers in English within the last 10 years. Several databases were searched using a combination of Medical Subject Heading (MeSH) phrases and keywords related to heart failure, loop diuretics, and thiazide diuretics. The search yielded 948 references, and after screening titles, abstracts, and full-text papers, eight final studies (five observational studies and three randomized control trials) were included in the review.

Based on the findings of this systematic review, there is substantial evidence to endorse the efficacy of combination diuretic therapy of loop and thiazide diuretics in augmenting diuresis and enhancing outcomes for patients who exhibit insufficient responses to single-agent diuretics. Additionally, the review provides valuable insights about the timing and type of diuretics to use, helping clinicians make informed therapeutic decisions. However, to ensure patient safety and well-being, it is imperative to take into account the potential for electrolyte disturbances and impacts on renal function, necessitating diligent and vigilant monitoring as well as effective management strategies.

In light of these findings, further research is warranted to optimize the dosing regimens and to delve deeper into the long-term safety and efficacy of combination therapy. Such research endeavors will undoubtedly contribute to refining treatment approaches and advancing patient care in the field of HF management.

## Introduction and background

Congestive heart failure (CHF) is a significant public health concern, affecting more than 64 million people worldwide, including 6.2 million cases in the US [1,2]. Among patients over the age of 65, CHF is the most common reason for hospitalization, characterized by acute decompensation episodes caused by volume overload and increased filling pressures, which require more intensive hospital treatment [3,4]. Intravenous (IV) loop diuretics are still a crucial part of acute decompensated heart failure (ADHF) therapy, as they can effectively relieve congestive symptoms and restore euvolemia, despite not having an established survival benefit [5-8]. According to Acute Decompensated Heart Failure National Registry (ADHERE) data, IV loop diuretics are administered to 88% of hospitalized ADHF patients [8-11].

Loop diuretics function by blocking the sodium-potassium-chloride co-transporter in the ascending loop of Henle, leading to the excretion of excess sodium and a strong aquaretic effect. This is a critical site for sodium reabsorption in the nephron [8,12]. Although loop diuretics are commonly used in treating ADHF, prolonged use may lead to drug resistance in heart failure (HF) patients [8,13,14]. While the Diuretic Optimization Strategies Evaluation (DOSE) study has evaluated early diuretic approaches in ADHF patients, there is limited information to guide the management of patients resistant to loop diuretics and experiencing refractory volume overload [15,16].

There are various explanations for why HF patients experience fluid overload that does not respond to treatment with loop diuretics. The most widely accepted explanation involves the way sodium retention works in HF and how the kidneys react to diuretics [4,17]. When loop diuretics are used, there may be a short-term increase in sodium retention after the diuretic has stopped working, known as the “post-diuretic effect". Long-term use of loop diuretics can also lead to renal adaptation, which involves the gradual enlargement and increased function of the distal nephron, resulting in elevated local sodium reabsorption. This "braking phenomenon" significantly reduces the effectiveness of loop diuretics [4,18,19]. Determining how much diuretic resistance a patient is experiencing is often based on a combination of subjective assessments of efficacy and objective measures like urine output and natriuresis. However, no standard definition of diuretic resistance can be consistently used at the bedside based on scientific evidence [20,21].

Sequential nephron blockade is another method used in medical practice for managing loop diuretic resistance in HF patients. This approach involves combining different diuretic classes such as loop and thiazide diuretics, which act on the distal convoluted tubule, and aldosterone antagonists which act on the collecting duct [22,23]. This combination therapy of loop and thiazide diuretics is believed to prevent or decrease sodium retention that occurs after loop diuretic treatment due to the longer half-lives of thiazides to loops [24,25]. Thiazides may enhance the effect of loop diuretics by inhibiting sodium reabsorption in the distal convoluted tubule and blocking the compensatory response that occurs with chronic exposure to loop diuretics [17,25]. However, despite the widespread use of combination diuretic therapy, there is little published research to guide clinicians in choosing the right timing and type of diuretics. This systematic review evaluates the effectiveness and safety of combination diuretic therapy with thiazide diuretics in refractory HF. By synthesizing available evidence, we aim to offer clinicians valuable insights into the potential benefits and limitations of this therapeutic strategy, contributing to optimized treatment approaches.

## Review

Methodology

The Preferred Reporting Items for Systematic Reviews and Meta-Analyses (PRISMA) 2020 guidelines were followed in this systematic review [26].

Eligibility Criteria

To select relevant studies for the review, certain criteria were established, including that the research should be conducted on human subjects and published in English as free full-text papers within the last 10 years (2013-2023). Additionally, the study population should consist of adults of both genders aged 18 years or older. However, articles published before 2013 or in languages other than English, articles involving patients under 18 years old, abstracts, conference papers, editorials, translated works, and gray literature were excluded from the study [27].

Search Strategy and Databases

We conducted a search for relevant publications on the effectiveness of combination diuretic therapy in HF using several databases such as PubMed/Medline, PubMed Central (PMC), Google Scholar, and Science Direct. To find the relevant papers, we used a Boolean approach that combined Medical Subject Heading (MeSH) phrases with keywords such as Heart Failure, Loop Diuretic, Furosemide, Thiazide, and Metolazone. The specifics of the search strategy are listed in Table 1. We organized the references into groups and removed duplicates using the EndNote reference manager. We then excluded irrelevant studies based on their titles and abstracts and finally examined full-text papers for further exclusion [27].

**Table 1:** Details Regarding the Search Strategy Implemented, Including the Specific Filters PubMed Central (PMC); Medical Subject Heading (MeSH); Heart Failure with reduced Ejection Fraction (HFrEF)

Search Strategy	Database Used	Number of Research Papers
("Heart Failure/classification"[Majr] OR "Heart Failure/complications"[Majr] OR "Heart Failure/diagnosis"[Majr] OR "Heart Failure/diagnostic imaging"[Majr] OR "Heart Failure/drug therapy"[Majr] OR "Heart Failure/etiology"[Majr] OR "Heart Failure/pathology"[Majr] OR "Heart Failure/physiopathology"[Majr] OR "Heart Failure/therapy"[Majr] ) AND ( "Furosemide/administration and dosage"[Majr] OR "Furosemide/adverse effects"[Majr] OR "Furosemide/metabolism"[Majr] OR "Furosemide/pharmacokinetics"[Majr] OR "Furosemide/pharmacology"[Majr] OR "Furosemide/therapeutic use"[Majr] OR "Furosemide/urine"[Majr] ) ( "Sodium Potassium Chloride Symporter Inhibitors/administration and dosage"[Majr] OR "Sodium Potassium Chloride Symporter Inhibitors/adverse effects"[Majr] OR "Sodium Potassium Chloride Symporter Inhibitors/pharmacokinetics"[Majr] OR "Sodium Potassium Chloride Symporter Inhibitors/pharmacology"[Majr] OR "Sodium Potassium Chloride Symporter Inhibitors/therapeutic use"[Majr] OR "Sodium Potassium Chloride Symporter Inhibitors/urine"[Majr] ) AND ( "Metolazone/administration and dosage"[Majr] OR "Metolazone/adverse effects"[Majr] OR "Metolazone/metabolism"[Majr] OR "Metolazone/pharmacokinetics"[Majr] OR "Metolazone/pharmacology"[Majr] OR "Metolazone/therapeutic use"[Majr] OR "Metolazone/urine"[Majr] ) OR ( "Thiazides/administration and dosage"[Majr] OR "Thiazides/adverse effects"[Majr] OR "Thiazides/metabolism"[Majr] OR "Thiazides/pharmacokinetics"[Majr] OR "Thiazides/therapeutic use"[Majr] OR "Thiazides/urine"[Majr] )	PubMed	157
HFrEF[All Fields] AND ("sodium potassium chloride symporter inhibitors"[All Fields] OR "sodium potassium chloride symporter inhibitors"[MeSH Terms] OR ("sodium"[All Fields] AND "potassium"[All Fields] AND "chloride"[All Fields] AND "symporter"[All Fields] AND "inhibitors"[All Fields]) OR "sodium potassium chloride symporter inhibitors"[All Fields] OR ("loop"[All Fields] AND "diuretic"[All Fields]) OR "loop diuretic"[All Fields]) AND ("thiazides"[MeSH Terms] OR "thiazides"[All Fields] OR "thiazide"[All Fields]) AND ("open access"[filter] AND "2013/04/22"[PDat] : "2023/04/19"[PDat])	PMC	273
allintitle: (Heart failure) AND (Loop OR Furosemide) AND (Thiazide OR Metolazone)	Google Scholar	11
HFrEF AND Loop Diuretic OR Furosemide AND Thiazide OR Metalozone	Science Direct	507

Results

Study Selection and Quality Evaluation

**Figure 1:**
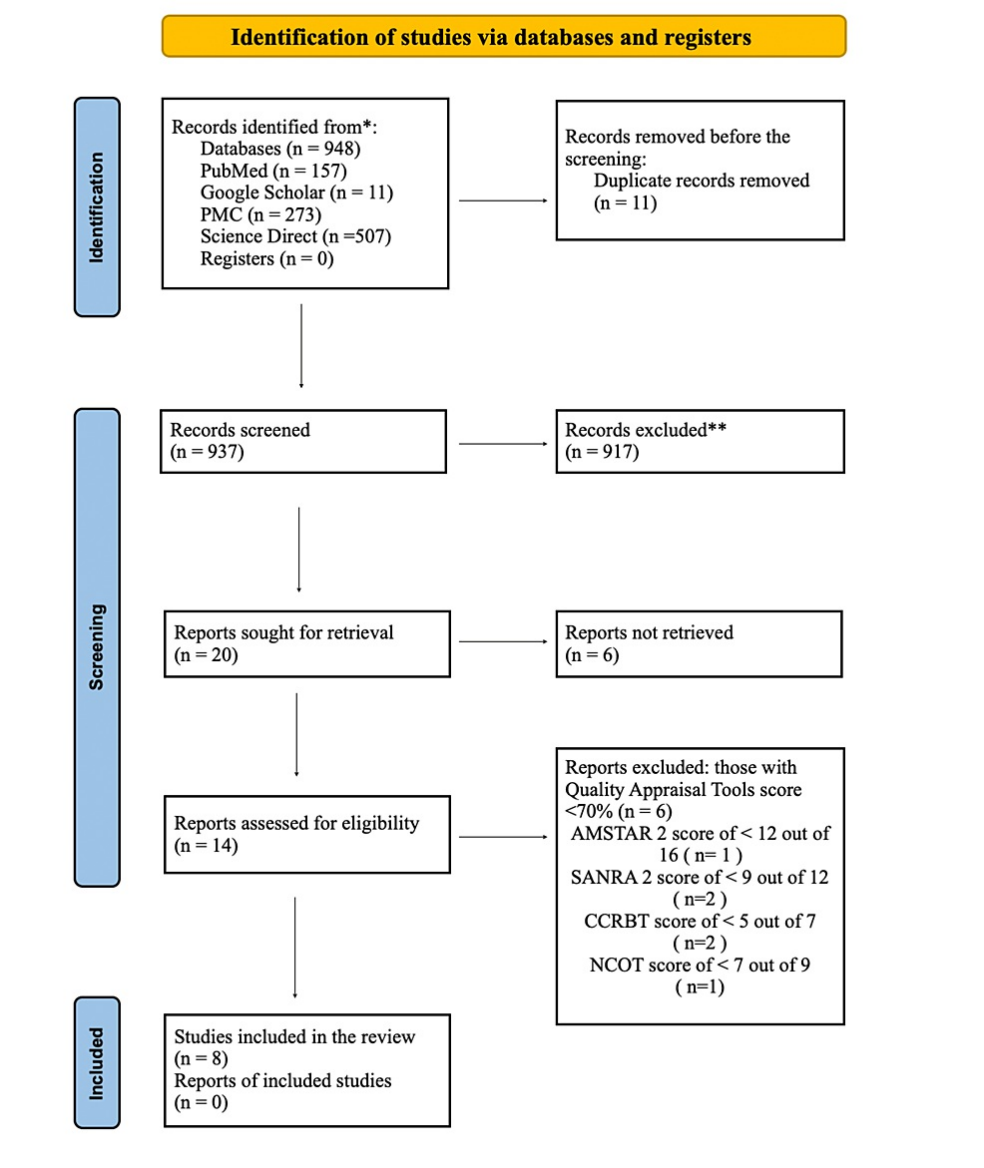
PRISMA Flowchart Assessment of Multiple Systematic Reviews (AMSTAR 2); Scale for the Assessment of Narrative Review Articles 2 (SANDRA 2); Cochrane Collaboration Risk of Bias Tool (CCRBT); New Castle Ottawa Tool (NCOT); PRISMA (Preferred Reporting Items for Systematic Reviews and Meta-Analyses)

Four databases, including PubMed, PubMed Central, Science Direct, and Google Scholar, yielded 948 references before April 20, 2023. Eleven duplicates were taken out after aggregating them. The remaining 937 records were reduced to 20 by the removal of 917 irrelevant records as a consequence of a title and abstract screening. Following the thorough full-text screening of the remaining 20 references, six records were eliminated, leaving us with just 14 of them. Then, using instruments created especially for each type of study, a quality assessment was carried out. Eight final studies with a score of more than 70% were included after the evaluation. Five observational studies and three randomized control trials (RCTs) made up the final research study [27]. A flow diagram representing the search selection is shown in Figure 1.

Study Characteristics

A comprehensive overview and detailed discussion of the quality assessment process and the specific tools utilized to evaluate the chosen articles for the review are given in Table 2.

**Table 2:** An In-Depth Exploration of the Quality Assessment Process and Utilized Tools for Evaluating Chosen Articles Cochrane Collaboration Risk of Bias Tool (CCRBT); New Castle Ottawa Tool (NCOT); Randomized Control Trails (RCTs)

Quality Assessment Tool	Type of Study	Total Score	Accepted Score (>70%)	Number of Accepted Studies (#)
CCRBT	RCTs	7	5	3; Trullàs JC et al. (2016) [4], Cox ZL et al. (2020) [20], Piardi DS et al. (2021) [23].
NCOT	Observational Studies	9	7	5; Moranville MP et al. (2015) [8], Shulenberger CE et al. (2016) [16], Côté JM et al. (2021) [21], Ng TM et al. (2013) [25], Goyfman M et al. (2017) [28].

The key features of the Observational Studies included in the analysis are given in Table 3.

**Table 3:** Analysis of the Principal Characteristics of the Incorporated Observational Studies Heart Failure (HF); Acute Decompensated Heart Failure (ADHD); Intensive Care Unit (ICU)

First Author, Year	Study Type	Inclusion Criteria	Sample Size	Outcome
Moranville MP (2015) [8]	Retrospective Cohort	The study included adult patients, aged 18 years and above, who had moderate to severe kidney dysfunction with a creatinine clearance between 15-50 ml/min. These patients were admitted to the hospital with a diagnosis of acute decompensated heart failure (ADHF), as indicated by specific ICD-9 codes. Initially, they were treated with intravenous furosemide alone, and after at least 72 hours, their treatment was intensified by adding either Metolazone or chlorothiazide in combination with furosemide.	55	In patients hospitalized with ADHF, renal insufficiency, and diuretic resistance receiving add-on thiazide-type diuretic treatment, there was no statistically significant difference in efficacy or safety between PO metolazone and IV chlorothiazide.
Shulenberger CE (2016) [16]	Retrospective Cohort	The study included patients aged 18 to 89 years who were admitted to the University of Maryland Medical Center in Baltimore, MD, between 2005 and 2015. These patients were diagnosed with Acute Decompensated Heart Failure (ADHF) and exhibited resistance to loop diuretics, specifically receiving 160 mg or more of intravenous furosemide during their hospitalization.	177	The effectiveness of oral metolazone was comparable to intravenous chlorothiazide in increasing urine output in patients with acute decompensated heart failure (ADHF) and resistance to loop diuretics. Moreover, both options demonstrated similar safety profiles regarding kidney function and electrolyte balance. Considering the substantial difference in cost between the two medications, these results indicate that oral metolazone could be a viable initial choice for this group of patients.
Côté JM (2021) [21]	Retrospective Cohort	Patients who were at least 18 years old and admitted to the Intensive Care Unit (ICU) for 24 hours or more and received intravenous loop diuretics at a dose greater than 1.0 mg per kg per day at any point during their ICU stay were considered eligible for inclusion in the study.	6358	The administration of continuous infusions of loop diuretics and the combination of thiazide or acetazolamide with loop diuretics resulted in a notable increase in urine production, ultimately leading to a reduction in fluid accumulation and subsequent weight reduction.
Ng TM (2013) [25]	Retrospective Cohort	Patients aged ≥18 years who received a minimum of 2 bolus doses of furosemide and then subsequently given a more intensive diuretic regimen typically reserved for those who are considered unresponsive to intermittent bolus furosemide.	242	The results indicate that treatment with F þ M or CIB was predictive of a greater change in hourly UO, independent of other variables.
Goyfman M (2017) [28]	Case Series	Patients admitted with signs and symptoms of AHF which was determined by the on-service attending physician using the Framingham criteria for Heart failure (HF).	17	Majority of the patients showed significant improvement from fluid overload signs and symptoms, with minimal fluctuations in serum electrolytes and creatinine levels.

Table 4 presents the key features of the RCTs included in the analysis.

**Table 4:** Analysis of the Key Features of the Randomized Clinical Trials in the Review Randomized Clinical Trial (RCT); Hydrochlorothiazide (HCTZ); Acute Heart Failure (AHF); Acute Decompensated Heart Failure (ADHF)

First Author, Year	Study Type	Inclusion Criteria	Sample Size (dropouts)	Intervention	Outcomes
Trullàs JC (2016) [4]	RCT	Individuals aged 18 years or older with a documented history of chronic heart failure, regardless of the underlying cause or ejection fraction, who are admitted due to acute decompensation, and have been receiving oral loop diuretic treatment for a minimum of 1 month before randomization will be included in the study.	304	Participants will be assigned randomly to receive either hydrochlorothiazide (HCTZ) or a placebo for 5 days, with an equal distribution ratio between the two groups. The dosage of the medication will be adjusted based on the individual's renal function. Following hospital discharge, all patients will be monitored for 90 days. If a patient is discharged before day 4, the necessary assessments will be conducted at outpatient clinics or heart failure units.	The main goals of the study are to examine the impact of adding a thiazide-type diuretic (HCTZ) to a loop diuretic on weight loss and the level of dyspnea reported by patients. The results of this trial will yield significant insights and have a substantial influence on treatment approaches and future research endeavors concerning these individuals.
Cox ZL (2020) [20]	RCT	Patients who were admitted to the hospital with hypervolemic acute heart failure (AHF) complicated by intravenous loop diuretic resistance were included in the study.	60(53)	The patients were randomly assigned in equal proportions of 1:1:1 to three different treatment groups: 1) taking oral metolazone at a dose of 5 mg twice daily, 2) receiving intravenous chlorothiazide at a dose of 500 mg twice daily, or 3) taking oral tolvaptan at a dose of 30 mg once daily. These treatments were added to their existing loop diuretic therapy, and the assigned treatment was administered for a maximum duration of 48 hours.	No significant disparities in the amount of weight loss were observed when either metolazone, intravenous chlorothiazide, or tolvaptan was introduced alongside high-dose intravenous loop diuretic medications in patients with acute heart failure (AHF) and diuretic resistance.
Piardi DS (2021) [23]	RCT	Individuals aged 18 years or older, diagnosed with acute decompensated heart failure (ADHF), admitted to the emergency department within the last 18 hours, with endogenous creatinine clearance greater than 30 mL/min, serum potassium levels between 3.5 and 5.1 mEq/L, and displaying signs of congestion will be included in the study.	51(4)	The patients who participated in the study were randomly assigned to one of two groups: HCTZ 50 mg or a placebo, both administered orally. The dosage of intravenous furosemide and any other supplementary treatments were determined by the patient's medical team based on their discretion.	The inclusion of HCTZ as an adjunct to the standard treatment for patients with acute decompensated heart failure (ADHF) did not show a statistically significant decrease in weight during the initial three days of hospitalization. However, upon adjusting the dosage of intravenous furosemide, the addition of 50 mg of HCTZ resulted in a statistically significant reduction in weight for every 40 mg of intravenous furosemide administered.

Discussion

This systematic review is aimed to evaluate the efficacy of combination diuretic therapy in refractory HF patients. Our analysis included a comprehensive search of electronic databases and relevant sources to identify eligible studies. The inclusion criteria consisted of RCTs and observational studies that assessed the effects of combination diuretic therapy in refractory HF patients.

Understanding Refractory Fluid Overload in HF

HF is a complex clinical syndrome characterized by impaired cardiac function leading to the inability of the heart to meet the body's demands for oxygen and nutrients. Loop diuretics, such as furosemide, are commonly used as the first-line treatment for fluid overload in HF. However, in some cases, loop diuretic mono-therapy may not effectively remove the excess fluid, leading to refractory fluid overload.

Refractory fluid overload refers to a condition in which HF symptoms persist despite receiving appropriate medical treatment, including diuretic therapy [29]. It happens when the body's sensitivity to diuretics is compromised, leading to decreased natriuresis and diuresis, which restricts the likelihood of reaching euvolemia [17]. To overcome this, the use of combination diuretic therapy, involving the addition of a second diuretic agent to loop diuretics such as thiazide diuretic, has been proposed as a strategy to enhance diuresis and improve fluid overload management in refractory HF patients.

The Concept of Combination Therapy with Thiazide Diuretics

Thiazide diuretics are a class of drugs that work by inhibiting the reabsorption of sodium in the distal convoluted tubule of the nephron [29]. By blocking sodium reabsorption, thiazide diuretics promote the excretion of sodium and water, leading to increased urine production and diuresis. The three most commonly used thiazide diuretics are hydrochlorothiazide (HCTZ), chlorothiazide, and metolazone [29].

The combination therapy of loop diuretics with thiazide diuretics aims to achieve a synergistic effect by targeting different segments of the nephron. Loop diuretics primarily act on the ascending loop of Henle, while thiazide diuretics exert their effects in the distal convoluted tubule [30]. This "sequential nephron blockade" allows for enhanced diuresis and sodium excretion by targeting multiple sites involved in renal sodium reabsorption. By combining these two classes of diuretics, the overall effectiveness of diuresis can be increased, particularly in cases of refractory fluid overload [30].

Evidence Supporting the Efficacy of Combination Therapy

In patients admitted with ADHF and excessive fluid accumulation, clinical guidelines recommend the prompt administration of IV loop diuretics at doses that match or surpass the total daily dose before admission [7]. When patients continue to experience congestive symptoms despite escalating loop diuretic requirements, the use of sequential nephron blockade with a thiazide-type diuretic can be considered a viable therapeutic strategy to enhance diuresis [16]. Both the American and European Heart Failure Guidelines now acknowledge that thiazide diuretics may be added to loop diuretics when diuresis remains inadequate even after increasing the loop diuretic doses [31,32].

Ng TM et al. conducted a retrospective study comparing the doses and effects of IV furosemide alone versus continuous infusion furosemide combined with metolazone. The study found that the median dose of intravenous furosemide was half of the continuous infusion dose. However, despite the lower daily dose, the combination with metolazone resulted in a 127% greater hourly diuresis [25]. These findings are consistent with previous literature indicating that the combination of thiazide and loop diuretics can enhance diuresis in patients who are resistant to a single agent. Additionally, a large randomized trial involving 33 patients with severe HF demonstrated that the addition of metolazone or bendrofluazide led to a diuretic response in 93% of the patients [33].

Hospitalizations for ADHF have garnered increasing attention both in research and political spheres due to their rising frequency and impact on healthcare resources [8]. A combined analysis of the DOSE and the Cardio-renal Rescue Study in Acute Decompensated Heart Failure (CARRESS-HF) trials revealed that more than one-third of patients remained congested upon discharge from the initial hospitalization [15]. Extensive evidence links the degree of volume removal achieved during ADHF hospitalizations with improved outcomes [34-41]. However, approximately 30% of AHF hospitalizations fail to achieve clinical decongestion by discharge, leading to higher mortality rates and increased re-hospitalization rates among Medicare patients [42-44].

To address this challenge, Goyfman M et al. conducted a case series involving 17 patients. They implemented an aggressive combination therapy of a diuretic and an aquaretic regimen, utilizing furosemide, metolazone, and spironolactone within an average of four days from the presentation. The mean duration of diuretic therapy was less than four days, while the mean daily urine output exceeded four liters. Importantly, this approach was associated with stable kidney function, hemodynamic parameters, and electrolyte levels [28]. The case series highlights the potential of combination diuretic therapy to facilitate rapid diuresis of a significant volume of fluid within a short timeframe. Ultimately, such an approach may help prevent prolonged hospital stays and frequent readmissions.

A more favorable diuretic response at the initiation of ADHF therapy has been linked to improved outcomes, including reduced symptoms, enhanced quality of life [15], decreased congestion [45], fewer admissions [46], and lower mortality rates [47]. Pardi DS et al., in a randomized placebo-controlled trial, supported this notion by incorporating HCTZ 50 mg per day into the standard treatment of ADHF patients. This intervention resulted in a weight loss of 0.7 kg per day greater than the placebo group, with an additional effect of 0.4 kg per day weight loss for every 40 mg of IV furosemide used [23]. These findings align with previous research conducted by Valente [47].

Other diuretics have also been investigated in the context of ADHF [23]. Cox ZL et al. conducted a study to explore the efficacy of a non-thiazide diuretic that acts distally to the loop of Henle in addressing cases of diuretic resistance (DR) occurring in HF. The mechanism of action of tolvaptan involves blocking the vasopressin-2 channel in the collecting duct, which results in a reduction in water absorption through aquaporin channels. Interestingly, tolvaptan exhibited a paradoxical trend of increased urine output but less weight loss compared to metolazone, although neither outcome reached statistical significance. One potential explanation for this discrepancy could be attributed to heightened thirst and unrecorded fluid intake, despite adherence to fluid restrictions [20].

Initial data showed promising results for the use of spironolactone as a diuretic, indicating its potential for reducing signs of congestion and lowering levels of natriuretic peptides [48]. Additionally, spironolactone was found to be a predictor of diuretic response [47]. However, the Aldosterone Targeted Neurohormonal Combined with Natriuresis Therapy in Heart Failure trial (ATHENA-HF), which evaluated the addition of 100 mg of spironolactone to the standard treatment of ADHF, did not confirm the previous findings. There was no reduction in natriuretic peptides, nor was there any additional weight loss compared to the placebo group [49].

Furthermore, the combination of acetazolamide and loop diuretics demonstrated similar efficacy to the more commonly used thiazide-loop-diuretic combination. However, this combination resulted in reduced serum bicarbonate and pH while increasing serum chloride levels [50]. Such an effect can be advantageous in certain cases, particularly for patients with severe metabolic alkalosis, as it may promote the weaning of mechanical ventilation [51,52].

Comparative Efficacy of Thiazide Diuretics

In refractory HF, the selection of a thiazide diuretic, such as HCTZ, metolazone, or chlorothiazide, would depend on various factors, including the patient's characteristics, medical history, response to previous treatments, and the specific goals of therapy. Some of the points to be considered are:

Pharmacokinetic properties: Thiazide diuretics exhibit variations in their pharmacokinetic characteristics, including differences in duration of action and potency. For instance, in certain cases, the IV formulation of chlorothiazide provides an advantage, especially for individuals experiencing significant gastrointestinal edema or those with variable absorption or the inability to take oral medications. However, there are concerns about the effective filtration of chlorothiazide through the glomerulus to reach its intended site of action in patients with renal insufficiency [53-55]. On the other hand, metolazone is believed to be less impacted by impaired glomerular filtration, but it may encounter challenges regarding reduced bioavailability in patients with notable gastrointestinal edema [53,55,56]. These observations align with the findings of Cox ZL et al., who noted that due to its slow gastrointestinal absorption and long half-life, metolazone could demonstrate equivalent efficacy to IV chlorothiazide only after 48 hours of administration [20].

Potency and dosing: The effectiveness and dosing of thiazide diuretics can vary among individuals. A retrospective study examined the impact of adding either oral HCTZ or IV chlorothiazide to loop diuretic therapy in 82 patients with ADHF. The study found that chlorothiazide demonstrated superior results. Within 24 hours of administration, patients receiving chlorothiazide experienced a significant improvement in urine output compared to those receiving HCTZ. Specifically, the mean increase was 1786 ± 1737 mL with chlorothiazide versus 934 ± 943 mL with HCTZ (p=0.005) [57].

According to HF guidelines, the recommended starting doses for chlorothiazide and metolazone are 250 mg and 2.5 mg, respectively with adjustments based on individual needs [6,7]. However, a study conducted by Moranville MP et al. reported that during a 72-hour study period, the median total amount of thiazide-type diuretic used as an add-on therapy was 2500 mg for chlorothiazide and 7.5 mg for metolazone. Based on this estimation patients in the chlorothiazide group received around three times more thiazide diuretic compared to those treated with metolazone. Interestingly despite the dosage, patients who received metolazone achieved a greater net urine output at 72 hours compared to those who received chlorothiazide. Furthermore, a higher percentage of patients in the metolazone group achieved a minimum of 3000 mL of net urine output within the 72-hour timeframe [8]. This finding aligns with a study conducted by Shulenberger CE et al. They suggested that even though ADHF patients may have reduced bioavailability the diuretic effects of metolazone can still be effective at lower concentrations due to their synergistic properties [16].

Balancing cost and care: Balancing economic considerations with patient care is crucial in the management of HF, particularly when choosing between different medication options. One aspect that often gets overlooked is the cost difference between IV chlorothiazide (at doses of 250 or 500 mg) and oral (PO) metolazone (at doses of 2.5 or 5.0 mg). It is worth noting that a single IV dose of chlorothiazide 500 mg is significantly more expensive averaging $348.00 compared to a PO dose of metolazone 5 mg, which costs approximately $1.51 [58,59].

Considering the costs associated with the management of HF which is recognized as the diagnosis-related group (DRG) with the highest expense, estimated at 20 million dollars annually, it becomes imperative to identify strategies for cost reduction while ensuring the quality of patient care [60]. According to the study conducted by Shulenberger CE et al., it was found that metolazone should be prioritized as the therapy of choice for patients experiencing ADHF. Chlorothiazide on the other hand should be reserved for individuals who do not respond adequately to metolazone or face challenges with oral treatment feasibility [16].

Safety Concerns of Combination Therapy

Combination diuretic therapy is commonly used in managing HF to overcome resistance and achieve optimal fluid balance. However, this treatment approach does come with some safety considerations. When it comes to the safety concerns of combination therapy, several factors need to be addressed.

Electrolyte disturbances: One significant safety concern associated with combining thiazide diuretics in therapy is the potential for electrolyte disturbances. Thiazide diuretics function by blocking the reabsorption of sodium in the kidney's distal tubules leading to increased excretion of sodium, water, and potassium. While this mechanism effectively reduces volume overload it also increases the risk of hypokalemia and hyponatremia. Supporting this observation, studies by Ng TM et al. have reported an incidence of hyponatremia and hypokalemia in patients receiving a combination of furosemide and metolazone compared to those on continuous infusion furosemide [25]. In another study by Côté JM et al., adding HCTZ to patients in the ICU was associated with an increased occurrence of hypokalemia [21]. Hypokalemia which refers to low levels of potassium can negatively affect cardiac function raising the risk of arrhythmias and sudden cardiac death. Similarly, low sodium levels, known as hyponatremia, can lead to cognitive changes, seizures, and in some cases cerebral edema. Therefore, it is crucial to monitor electrolyte levels when using combination diuretic therapy with thiazides and take appropriate measures to prevent and manage these imbalances. However, upon comparing the incidence of electrolyte abnormalities among thiazide regimens in this review no statistical difference was found [8,25].

Renal function: When using a combination of thiazide diuretics in therapy an important consideration is the impact on kidney function. Thiazide diuretics have been known to cause a temporary decline in renal function especially in patients with preexisting kidney insufficiency. The exact mechanism behind this effect is not fully understood; however, it is believed to be related to the decrease in circulating volume and subsequent activation of compensatory mechanisms. It is important to note that these temporary changes, similar to the changes seen in the Diuretic Optimization Strategy Evaluation in Acute Decompensated Heart Failure (DOSE-HF) and Renal Optimization Strategies Evaluation (ROSE-HF) studies, do not indicate a true renal injury and typically resolve with proper monitoring and management [15,61]. Piardi DS et al. conducted a study where the addition of HCTZ, despite showing a greater change in creatinine levels, did not result in a difference in the onset of worsening renal failure when compared between the study groups [23]. Previous research has also shown that increased creatinine levels do not indicate a poor prognosis when patients have reduced congestion [28,62]. Moreover, a recent survey conducted among critical care physicians revealed that the majority (95%) considered it appropriate to continue diuretic therapy for fluid overload even if there are mild increases in creatinine or electrolyte and acid-base abnormalities [63]. Nevertheless, it is important to monitor renal function, including serum creatinine levels and urine output when using thiazide diuretics as part of combination therapy, especially in patients, with compromised kidney function.

Individual patient factors: The safety concerns associated with combination diuretic therapy with thiazide diuretics in refractory HF also depend on individual patient factors. Factors such as age, co-morbidities, concomitant medications, and overall fluid status can influence the risk of adverse effects. For instance, a positive fluid balance of more than 10% during an ICU stay has been associated with worsening kidney function and acute kidney injury (AKI) [64]. This highlights the importance of considering the patient's fluid status and adjusting diuretic therapy accordingly to maintain a balance between achieving diuresis and preventing fluid depletion. Additionally, the choice of the specific thiazide diuretic may also impact safety. Contrastingly, the studies in this review showed no significant difference in the occurrence of adverse effects among different thiazide regimens. This may have implications for diuretic efficacy and the occurrence of adverse effects. Therefore, individual patient factors should be carefully considered when selecting the appropriate thiazide diuretic and monitoring for safety concerns.

Patient education: Furthermore, patient education plays a vital role in promoting safety and minimizing risks. Patients should be informed about the potential side effects of thiazide diuretics, including the signs and symptoms of electrolyte disturbances, such as muscle weakness, fatigue, and irregular heart rhythms. They should be encouraged to report any concerning symptoms promptly. Moreover, healthcare professionals should emphasize the importance of medication adherence and inform patients about the need for regular follow-up appointments to monitor their response to therapy and adjust the treatment plan as necessary.

Limitations

Several shortcomings can be identified in the systematic review. For starters, it only considered papers published in English, which may create bias and ignore relevant studies published in other languages. Furthermore, by limiting inclusion to free full-text papers, there is a risk of publication bias favoring research with substantial results. Another disadvantage is that the search was limited to the last 10 years, potentially omitting older studies with useful insights. Exclusion criteria such as abstracts and conference papers may add bias and limit the comprehensiveness of the evaluation. Even though the search approach was thorough, there is still a risk that important research was overlooked. Due to subjectivity, the quality assessment and selection procedure may introduce bias, and omitting lower-scoring research may result in the loss of key insights. Synthesizing data from diverse studies poses challenges, and the absence of a meta-analysis restricts precise effect estimates and overall strength assessment of the evidence. In conclusion, while this systematic review follows the PRISMA 2020 guidelines and presents a comprehensive analysis of combination diuretic therapy in HF, it is important to consider the aforementioned limitations. Future research should aim to address these limitations to further enhance the understanding and applicability of combination diuretic therapy in the management of HF.

## Conclusions

In conclusion, combination diuretic therapy, particularly the addition of thiazide diuretics to loop diuretics, holds promise in the management of refractory fluid overload in HF patients. The concept of sequential nephron blockade, targeting different segments of the nephron, allows for enhanced diuresis and sodium excretion, addressing the persistent congestion and symptoms associated with refractory fluid overload. The evidence from various studies supports the efficacy of combination therapy in enhancing diuresis and improving outcomes in patients with inadequate response to single-agent diuretic therapy. The comparative efficacy of thiazide diuretics highlights the importance of considering individual patient factors, such as pharmacokinetic properties, potency, and dosing, in selecting the most appropriate thiazide diuretic for each patient. Additionally, cost considerations play a role in treatment decisions, with metolazone being a more cost-effective option compared to intravenous chlorothiazide.

While combination diuretic therapy can be effective, it is not without safety concerns, particularly related to electrolyte disturbances and renal function. Close monitoring of electrolyte levels and appropriate measures to prevent and manage electrolyte imbalances is crucial in ensuring patient safety. Overall, the evidence supports the use of combination diuretic therapy as a viable approach for managing refractory fluid overload in HF patients. Further research and well-designed clinical trials are needed to optimize the selection of thiazide diuretics, determine the most effective dosing regimens, and better understand combination therapy's long-term safety and efficacy in this patient population.

## References

[1] Savarese G, Becher PM, Lund LH, Seferovic P, Rosano GM, Coats AJ (2023). Global burden of heart failure: a comprehensive and updated review of epidemiology. Cardiovasc Res.

[2] Virani SS, Alonso A, Benjamin EJ (2020). Heart disease and Stroke Statistics-2020 update: a report from the American Heart Association. Circulation.

[3] Roger VL (2013). Epidemiology of heart failure. Circ Res.

[4] Trullàs JC, Morales-Rull JL, Casado J, Freitas Ramírez A, Manzano L, Formiga F (2016). Rationale and design of the “safety and efficacy of the combination of loop with thiazide-type diuretics in patients with decompensated heart failure (Clorotic) trial:” a double-blind, randomized, placebo-controlled study to determine the effect of combined diuretic therapy (loop diuretics with thiazide-type diuretics) among patients with decompensated heart failure. J Card Fail.

[5] Hunt SA, Abraham WT, Chin MH (2009). 2009 focused update incorporated into the ACC/AHA 2005 Guidelines for the diagnosis and management of heart failure in adults: a report of the American College of Cardiology Foundation/American Heart Association Task Force on practice guidelines: developed in collaboration with the International Society for Heart and lung transplantation. Circulation.

[6] Lindenfeld J, Albert NM, Boehmer JP (2010). HFSA 2010 comprehensive heart failure practice guideline. J Card Fail.

[7] Yancy CW, Jessup M, Bozkurt B (2013). 2013 ACCF/AHA guideline for the management of heart failure: a report of the American College of Cardiology Foundation/American Heart Association Task Force on Practice Guidelines. J Am Coll Cardiol.

[8] Moranville MP, Choi S, Hogg J, Anderson AS, Rich JD (2015). Comparison of metolazone versus chlorothiazide in acute decompensated heart failure with diuretic resistance. Cardiovasc Ther.

[9] Marik PE, Flemmer M (2012). Narrative review: the management of acute decompensated heart failure. J Intensive Care Med.

[10] Roger VL, Go AS, Lloyd-Jones DM (2011). Heart disease and stroke statistics-2011 update: a report from the American Heart Association. Circulation.

[11] Peacock WF, Costanzo MR, De Marco T, Lopatin M, Wynne J, Mills RM, Emerman CL (2009). Impact of intravenous loop diuretics on outcomes of patients hospitalized with acute decompensated heart failure: insights from the ADHERE registry. Cardiology.

[12] Majure DT, Teerlink JR (2011). Update on the management of acute decompensated heart failure. Curr Treat Options Cardiovasc Med.

[13] Jentzer JC, DeWald TA, Hernandez AF (2010). Combination of loop diuretics with thiazide-type diuretics in heart failure. J Am Coll Cardiol.

[14] Ellison DH (1991). The physiologic basis of diuretic synergism: its role in treating diuretic resistance. Ann Intern Med.

[15] Felker GM, Lee KL, Bull DA (2011). Diuretic strategies in patients with acute decompensated heart failure. N Engl J Med.

[16] Shulenberger CE, Jiang A, Devabhakthuni S, Ivaturi V, Liu T, Reed BN (2016). Efficacy and safety of intravenous chlorothiazide versus oral metolazone in patients with acute decompensated heart failure and loop diuretic resistance. Pharmacotherapy.

[17] Ellison DH (2001). Diuretic therapy and resistance in congestive heart failure. Cardiology.

[18] Kim GH (2004). Long-term adaptation of renal ion transporters to chronic diuretic treatment. Am J Nephrol.

[19] Loon NR, Wilcox CS, Unwin RJ (1989). Mechanism of impaired natriuretic response to furosemide during prolonged therapy. Kidney Int.

[20] Cox ZL, Testani JM (2020). Loop diuretic resistance complicating acute heart failure. Heart Fail Rev.

[21] Côté JM, Bouchard J, Murray PT, Beaubien-Souligny W (2021). Diuretic strategies in patients with resistance to loop-diuretics in the intensive care unit: a retrospective study from the MIMIC-III database. J Crit Care.

[22] Martens P, Nijst P, Mullens W (2015). Current approach to decongestive therapy in acute heart failure. Curr Heart Fail Rep.

[23] Piardi DS, Butzke M, Mazzuca AC (2021). Effect of adding hydrochlorothiazide to usual treatment of patients with acute decompensated heart failure: a randomized clinical trial. Sci Rep.

[24] De Bruyne LK (2003). Mechanisms and management of diuretic resistance in congestive heart failure. Postgrad Med J.

[25] Ng TM, Konopka E, Hyderi AF (2013). Comparison of bumetanide- and metolazone-based diuretic regimens to furosemide in acute heart failure. J Cardiovasc Pharmacol Ther.

[26] Page MJ, McKenzie JE, Bossuyt PM (2021). The PRISMA 2020 statement: an updated guideline for reporting systematic reviews. Int J Surg.

[27] Dawood SN, Rabih AM, Niaj A (2022). Newly discovered cutting-edge triple combination cystic fibrosis therapy: a systematic review. Cureus.

[28] Goyfman M, Zamudio P, Jang K, Chee J, Miranda C, Butler J, Wadhwa NK (2017). Combined aquaretic and diuretic therapy in acute heart failure. Int J Nephrol Renovasc Dis.

[29] McKee PA, Castelli WP, McNamara PM, Kannel WB (1971). The natural history of congestive heart failure: the Framingham study. N Engl J Med.

[30] Givertz MM, Postmus D, Hillege HL (2014). Renal function trajectories and clinical outcomes in acute heart failure. Circ Heart Fail.

[31] Yancy CW, Jessup M, Bozkurt B (2017). 2017 ACC/AHA/HFSA focused update of the 2013 ACCF/AHA guideline for the management of heart failure: a report of the American College of Cardiology/American Heart Association Task Force on clinical practice guidelines and the Heart Failure Society of America. Circulation.

[32] Ponikowski P, Voors AA, Anker SD (2016). 2016 ESC Guidelines for the diagnosis and treatment of acute and chronic heart failure: the Task Force for the diagnosis and treatment of acute and chronic heart failure of the European Society of Cardiology (ESC)Developed with the special contribution of the Heart Failure Association (HFA) of the ESC. Eur Heart J.

[33] Channer KS, McLean KA, Lawson-Matthew P, Richardson M (1994). Combination diuretic treatment in severe heart failure: a randomised controlled trial. Br Heart J.

[34] Barsuk JH, Gordon RA, Cohen ER, Cotts WG, Malkenson D, Yancy CW, Williams MV (2013). A diuretic protocol increases volume removal and reduces readmissions among hospitalized patients with acute decompensated heart failure. Congest Heart Fail.

[35] Costanzo MR, Guglin ME, Saltzberg MT (2007). Ultrafiltration versus intravenous diuretics for patients hospitalized for acute decompensated heart failure. J Am Coll Cardiol.

[36] Gheorghiade M, Vaduganathan M, Fonarow GC, Bonow RO (2013). Rehospitalization for heart failure: problems and perspectives. J Am Coll Cardiol.

[37] Thavendiranathan P, Yingchoncharoen T, Grant A, Seicean S, Landers SH, Gorodeski EZ, Marwick TH (2014). Prediction of 30-day heart failure-specific readmission risk by echocardiographic parameters. Am J Cardiol.

[38] Ambrosy AP, Pang PS, Khan S (2013). Clinical course and predictive value of congestion during hospitalization in patients admitted for worsening signs and symptoms of heart failure with reduced ejection fraction: findings from the EVEREST trial. Eur Heart J.

[39] Kociol RD, McNulty SE, Hernandez AF (2013). Markers of decongestion, dyspnea relief, and clinical outcomes among patients hospitalized with acute heart failure. Circ Heart Fail.

[40] Sharma GV, Woods PA, Lindsey N, O'Connell C, Connolly L, Joseph J, McIntyre KM (2011). Noninvasive monitoring of left ventricular end-diastolic pressure reduces rehospitalization rates in patients hospitalized for heart failure: a randomized controlled trial. J Card Fail.

[41] Pimenta J, Paulo C, Mascarenhas J, Gomes A, Azevedo A, Rocha-Gonçalves F, Bettencourt P (2010). BNP at discharge in acute heart failure patients: is it all about volemia? A study using impedance cardiography to assess fluid and hemodynamic status. Int J Cardiol.

[42] Chioncel O, Mebazaa A, Maggioni AP (2019). Acute heart failure congestion and perfusion status - impact of the clinical classification on in-hospital and long-term outcomes; insights from the ESC-EORP-HFA Heart Failure Long-Term Registry. Eur J Heart Fail.

[43] Lala A, McNulty SE, Mentz RJ (2015). Relief and recurrence of congestion during and after hospitalization for acute heart failure: insights from diuretic optimization strategy evaluation in acute decompensated heart failure (dose-AHF) and cardiorenal rescue study in acute decompensated heart failure (Caress-HF). Circ Heart Fail.

[44] Ross JS, Chen J, Lin Z (2010). Recent national trends in readmission rates after heart failure hospitalization. Circ Heart Fail.

[45] Rohde LE, Beck-da-Silva L, Goldraich L, Grazziotin TC, Palombini DV, Polanczyk CA, Clausell N (2004). Reliability and prognostic value of traditional signs and symptoms in outpatients with congestive heart failure. Can J Cardiol.

[46] Beck da Silva L, Mielniczuk L, Laberge M, Anselm A, Fraser M, Williams K, Haddad H (2004). Persistent orthopnea and the prognosis of patients in the heart failure clinic. Congest Heart Fail.

[47] Valente MA, Voors AA, Damman K (2014). Diuretic response in acute heart failure: clinical characteristics and prognostic significance. Eur Heart J.

[48] Ferreira JP, Santos M, Almeida S, Marques I, Bettencourt P, Carvalho H (2014). Mineralocorticoid receptor antagonism in acutely decompensated chronic heart failure. Eur J Intern Med.

[49] Butler J, Anstrom KJ, Felker GM (2017). Efficacy and safety of spironolactone in acute heart failure: the Athena-HF randomized clinical trial. JAMA Cardiol.

[50] Brown AJ, Cutuli SL, Eastwood GM, Bitker L, Marsh P, Bellomo R (2019). A pilot randomised controlled trial evaluating the pharmacodynamic effects of furosemide versus acetazolamide in critically ill patients. Crit Care Resusc.

[51] Heming N, Faisy C, Urien S (2011). Population pharmacodynamic model of bicarbonate response to acetazolamide in mechanically ventilated chronic obstructive pulmonary disease patients. Crit Care.

[52] Faisy C, Mokline A, Sanchez O, Tadié JM, Fagon JY (2010). Effectiveness of acetazolamide for reversal of metabolic alkalosis in weaning COPD patients from mechanical ventilation. Intensive Care Med.

[53] Asscher AW (1974). Treatment of frusemide resistant edema with metolazone. Clin Trials J.

[54] (2023). DIURIL® (chlorothiazide) Oral Suspension - Shared Salix. https://shared.salix.com/globalassets/pi/Diuril-pi.pdf.

[55] McMurray JJ, Adamopoulos S, Anker SD (2012). ESC Guidelines for the diagnosis and treatment of acute and chronic heart failure 2012: the task force for the diagnosis and treatment of acute and chronic heart failure 2012 of the European Society of Cardiology. Developed in collaboration with the Heart Failure Association (HFA) of the ESC. Eur Heart J.

[56] (2023). Product monograph Zaroxolyn (metolazone) 2.5 mg diuretic/antihypertensive. https://pdf.hres.ca/dpd_pm/00044056.PDF.

[57] Kissling KT, Pickworth KK (2014). Comparison of the effects of combination diuretic therapy with oral hydrochlorothiazide or intravenous chlorothiazide in patients receiving intravenous furosemide therapy for the treatment of heart failure. Pharmacotherapy.

[58] Thomson Thomson (2010). Red Book: Pharmacy's Fundamental Reference.

[59] Lau BD, Pinto BL, Thiemann DR, Lehmann CU (2011). Budget impact analysis of conversion from intravenous to oral medication when clinically eligible for oral intake. Clin Ther.

[60] Fida N, Piña IL (2012). Trends in heart failure hospitalizations. Curr Heart Fail Rep.

[61] Ahmad T, Jackson K, Rao VS (2018). Worsening renal function in patients with acute heart failure undergoing aggressive diuresis is not associated with tubular injury. Circulation.

[62] Metra M, Davison B, Bettari L (2012). Is worsening renal function an ominous prognostic sign in patients with acute heart failure? The role of congestion and its interaction with renal function. Circ Heart Fail.

[63] Silversides JA, McAuley DF, Blackwood B, Fan E, Ferguson AJ, Marshall JC (2020). Fluid management and deresuscitation practices: a survey of critical care physicians. J Intensive Care Soc.

[64] Bouchard J, Soroko SB, Chertow GM, Himmelfarb J, Ikizler TA, Paganini EP, Mehta RL (2009). Fluid accumulation, survival and recovery of kidney function in critically ill patients with acute kidney injury. Kidney Int.

